# The Evaluation of Therapeutic Residential Care for Adolescents and Young Adults in France: A Systematic Review

**DOI:** 10.3389/fpsyt.2021.609365

**Published:** 2021-05-20

**Authors:** France Hirot, Caroline Huas, Damien Durand, Nathalie Godart

**Affiliations:** ^1^Centre de Recherche en Épidémiologie et Santé des Populations, INSERM, UMR 1018, Université Paris-Saclay, Hôpital Paul Brousse, VillejuifCedex, France; ^2^Service hospitalo-universitaire de Santé Mentale de l'Adolescent et du Jeune Adulte, Fondation Santé des Etudiants de France, Paris, France; ^3^UFR Simone Veil-Santé, Université Versailles Saint-Quentin-en-Yvelines, Montigny-le-Bretonneux, France; ^4^Directeur national des études et de la pédagogie de la Fondation Santé des Etudiants de France, Paris, France; ^5^Inspecteur d'académie - inspecteur pédagogique régional, spécialité établissements et vie scolaire, Ministère de l'Education Nationale (French Minister of National Education), Paris, France

**Keywords:** adolescents, youth, therapeutic residential care, *soins-études*, schooling, studies

## Abstract

Early psychosocial rehabilitation of young people presenting mental disorders is a major challenge. In France, the therapeutic residential care called “*soins-études*,” combining care and educational provision, in the *Fondation Santé des Etudiants de France* (FSEF) can have a role in this rehabilitation. After recalling the history and the concept underpinning *soins-études* in psychiatry, we performed a systematic review of the literature based on the PRISMA statement *via* a search for quantitative studies on *soins-études* facilities. Eleven quantitative studies on 10 different samples of young people hospitalised in psychiatry in FSEF were identified between the opening of the first unit in 1956 and 2016. The young people involved were mostly aged 16–20 years, which reflects the curricula covered in the FSEF establishments. These young people generally presented severe chronic psychiatric disorders. Their previous care trajectory had lasted for more than 3 years and 24–55% of them had attempted suicide at least once. Their stays lasted more than 6 months. Depending on the severity of the disorders, 44–63% of the young people were considered to have improved at discharge. The contribution of *soins-études* appears valuable for these young people, since there was a clinical improvement for 54–74% of them 1–15 years after their hospitalisation, with resumption of schooling, professional training or entry into employment in 60–75% of the cases. These results are compared with data in the international literature concerning therapeutic residential care, and lines for future research are identified.

## Introduction

Most chronic psychiatric disorders have an onset before the age of 18 years (1). Psychiatric disorders have severe consequences (1) when their evolution is chronic, generating mental suffering, addictive and somatic comorbidities, social withdrawal, early deaths (somatic or from suicide), and considerable individual, familial and societal cost. Starting psychosocial rehabilitation at an early stage is crucial to limit these consequences (2). Care provision among young people is mainly ambulatory, but it can also require hospitalisation (3). Despite the concerns presently voiced as to the damaging effects of prolonged, inpatient treatment, this type of approach continues to be required (4), in particular when the persistence of symptoms and the need for care compromise schooling.

In France psychiatric therapeutic residential care known as “*soins-études*” were developed in order to enable inpatient care and schooling for severely ill adolescents and youg adults in one and the same place. This is one of the systems engaged in psychosocial rehabilitation (2), enabling the continuity of care and alongside the resumption of schooling. The young people receive global care suited to their needs, including psychiatric healthcare, maintenance or resumption of schooling and social rehabilitation within a group of peers, before discharge into the community.

### History and Concept

The model of residential treatment appeared in the 1950s to treat children and adolescents with psychiatric disorders (5). There were no distinctions between hospitals and other institutions, and the term residential centre gathered the institutions that oriented the daily life of children around psychodynamic and therapeutic principles and where childcare staff were to serve as primary therapeutic agents (5). The care was then described as therapy mediated by the environment, or “milieu therapy,” where treatment was ensured by the overall team and comprised daily interaction with the young people (5). It was viewed as a solution to neglect and mistreatment (5). With the community mental health movements, these extended inpatient treatment strategies involving separation between children and their parents raised concerns about effectiveness, child safety and costs (4). Better assessment of the care provided and better definition of the target population and of the modes of functioning were therefore required (4). In 2016, an international consensus defined therapeutic residential care as facilities providing multi-dimensional living environments designed to enhance or provide treatment, education, socialisation, support, and protection for young people with identified mental health needs in partnership with their families and in collaboration with a full spectrum of community-based formal and informal resources (4). However, therapeutic residential care gathers highly heterogeneous programs. Thus, the terms to describe “residential care” are indeed numerous: “congregate care,” “group care,” “children's homes,” “socio-pedagogical homes” etc. (4), and they can cover different modes of healthcare, as well as child welfare with socio-educational or judicial establishments.

In France, the *soins-études* system is a form of therapeutic residential care for adolescents and young adults. They are healthcare facilities where young people are referred by their psychiatrist for inpatient care when they have not responded well to other types of care. The young people are admitted for any psychiatric disorders starting in childhood or adolescence when all other types of treatments, including outpatient and day care or intensive treatment at home, have been tried. This particular situation does not enable normal school attendance. They are not placed by a child welfare system, they are hospitalised at their own or their parents' request (*soins-études* units are not children's homes) nor are *soins-études* units part of juvenile delinquency prevention. Young people who are admitted were often previously invested in their studies, and are motivated to continue but have dropped out because of their health problems. The system could be seen as a supported education program applied to inpatient care (6).

*Fondation Santé des Etudiants de France* (FSEF; French Student Health Foundation) was established by the *Union Nationale des Etudiants de France* (UNEF; National Union of Students of France). The FSEF first created establishments in which students could pursue their studies while at the same time receiving the long-term hospital care required by tuberculosis (7, 8). After the Second World War and following the arrival of the first antibiotics which improved the prognosis of tuberculosis, mental health appeared as the “number one health problem” in student circles (9). In 1953, the FSEF convert its *soins-études* system for students suffering from tuberculosis to mental disorders care (10). The aim of the FSEF was to avoid the marginalisation of young people on account of their pathology and to foster their social rehabilitation (11). FSEF psychiatric *soins-études* units were designed to take on students whose mental disturbances required hospital care, but were nevertheless compatible with the pursuit of some degree of academic work (8, 12). They were designed as open psychiatric facilities to avoid institutional withdrawal and the potentially iatrogenic effects (13). These were thus inpatient hospital facilities where the young people catered for could come and go freely in the daytime so as to favour links with the outside world (13–15). The students were in particular encouraged to attend courses in their original University and maintain their leisure activities outside the facilities (8, 16). Gradually, establishments catering for upper secondary school and later lower secondary school students were set up so as to provide care for younger people, at an earlier stage in their disorders (8, 14). Each year, more than 900 young people are hospitalised full time in *soins-études* in psychiatry within FSEF.

The *soins-études* are defined by the presence in all establishments of teachers from the national education system (French Minister of National Education), the units being attached as pedagogical annexes to the national authority (8, 13). The teachers provide pedagogical support to University students who cannot attend in their educational establishments and implement courses for secondary school (8, 16). Since the creation of these structures, care provision is the result of close collaboration between healthcare professionals and teachers (8, 12, 13). Schooling becomes an extension of the treatment environment, and treatment and educational activities are highly integrated (17–22). This system enables young people to be confronted with the realities of academic requirements, but in a venue where the teaching is highly individualised and flexible, so as to favour the mobilisation of their resources and skills (15, 16, 23).

Today, 11 FSEF establishments cater for young people with mental disorders across France. Rather than providing assistance in passing examinations, the aims of *soins-études* are to ensure the rehabilitation of the student and to re-establish the student's autonomy (14, 15, 23), which is close to description of psycho-social rehabilitation, which entails a range of methods aiming to help people with mental disorders to regain a satisfactory standard of living and adaptation in relation to their expectations. The care provision is global, both medical, pedagogical and social, and centred on the young person in collaboration with his or her family (14, 16, 24). The admission of young people is based on medical indications for the pursuit of psychiatric care in hospital environment (17, 19). Curricula are coordinated with the therapeutic project and are considered as one of the therapeutic mediations offered (17, 18, 20, 25–27). The care is managed along the lines of hospital psychiatric care, including regular individual and family psychiatric consultations, interviews with nurses, as well as specific psychotherapies and psychotropic medication if required. In addition, group activities are proposed, deploying various therapeutic mediations (bodily approaches, artistic and cultural or social approaches), support groups, and psycho-education (17–19). S*oins-études* are inpatient programs, but adolescents return to their family home during weekends and half of the school holidays. The parents or carers are regularly involved in appointments in order to design the therapeutic project. The project is co-constructed with the young person, his or her family, the care professionals, the teachers and the various structures upstream and downstream (17–20, 25, 28, 29). The articulation between education and healthcare provides coherence (17, 19, 20, 22, 30). The aim is to enable the young person to be active in a project suited to his or her present situation (16, 20, 27, 28).

The therapeutic residential care is often being called into question for reasons of funding (4). Similarly, *soins-études* have a high cost, and their cost effectiveness could be questioned in the current economical context. However, from a clinical perspective, there is a small but substantial group of adolescents and young adults with severe disorders that benefits from this type of mental health care. *Soins-études* still appear to be needed when others types of care have not been sufficient. Thus they are recommended in French national guidelines for adolescents and young adults with psychiatric disorders (31–33). Indeed this type of care can avoid damages for social integration that exist in long lasting acute psychiatric hospitalisation. Nevertheless more evaluation is needed. As in all complex intervention evaluation, this assessment begin with a literature review. The aim of this study is to summarise all the existing reports on efficacy of *soins-études* up until now by an exhaustive review of the literature.

## Method

We carried out a review of the literature based on the PRISMA statement (34), with a systematic search of international (Pubmed) and French scientific databases (Cairn, Pascal et Francis). The French term “*soins-études*” could not be translated adequately into English. Several terms were explored, and the phrase “residential treatment,” gathering various types of healthcare, was retained. Given the small number of studies retrieved, we decided to complete the bibliographic search on the ScienceDirect database, managed by the publisher Elsevier. This Anglo-Dutch publishing group acquired the Publisher Masson, the main scientific publisher in France, in 2005. Despite the risk of publication bias, this search enabled us to identify numerous relevant references, since most French publications on *soins-études* were published in French. The search terms used in the different databases were as follows: “residential treatment” AND adolescent AND France AND psychiatric; “residential treatment” AND “Fondation Santé des Etudiants de France” on Pubmed; “*soin études*” AND psychiatrie AND adolescent; “*soins-études*” AND “Fondation Santé Etudiants de France” in Cairn and Pascal et Francis; all of these terms, in both French and English, on ScienceDirect.

The search was then completed in libraries and documentation centres: the Henry Ey medical library in Ste Anne Hospital, Paris, the Bibliothèque InterUnivesitaire de Santé (which in particular gathers all the medical theses in France), the Conservatoire des Mémoires d'Etudiants (Conservatory of Student Memories; online), the Bibliothèque de Documentation Internationale Contemporaine in Nanterre University (a library-museum covering the history of the twentieth and twenty-first centuries), and the Bibliothèque Sigmund Freud run by the Société Psychanalytique de Paris. We also consulted the archives of the FSEF general management, those stored by the National Director of Studies, and archives in the FSEF clinical libraries.

The data collection was completed by interviews with psychiatrists and physicians formerly in charge of FSEF clinics so as to identify further references. The search was also extended to caregivers and teachers working in FSEF. Finally we performed a manual search by analysing the bibliographies of the different documents retrieved so as to identify any references that had not been previously found.

**Figure 1:**
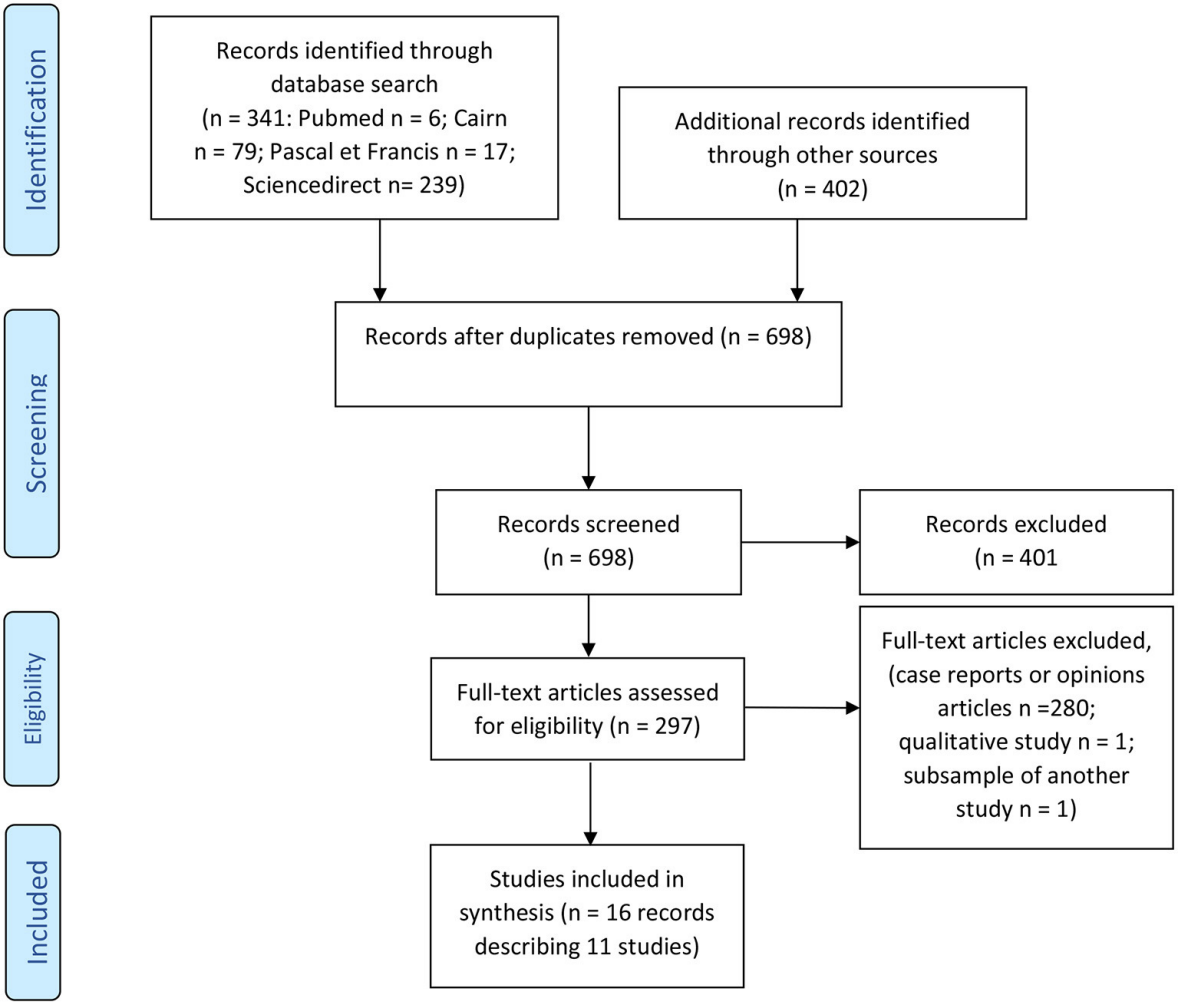
Flowchart.

The selection process was performed in two stages (34) by two of the authors (FH and NG), first from the titles and abstracts, then from the full text to assess eligibility (See Flowchart in Figure 1). All studies analysing quantitatively the population catered for in *soins-études* in psychiatry, and the evolution observed among young people in the course hospitalisation in FSEF *soins-études* and thereafter, since the opening of the first unit (02/04/1956 up to 31/01/2019), were included. Studies excluded were those that were case studies, qualitative studies and opinion papers (see Figure 1). Due to the very small number of studies, all quantitative studies were included in this review whether they were published or not (if they were academic theses).

Three hundred and forty-one records were identified through the database search and 402 through libraries, documentation centres and manual search. After the first screening, 297 full-text articles were assessed for eligibility. Full text screening excluded 280 case reports or opinions articles and two medical theses carried out within FSEF: the work by Perrier (35) used a qualitative method on a sample returned to later by Pages (36); the work by Bié (37) was considered to duplicate a population described in the work by Condé-Diaz (38) without providing new information. Sixteen records describing 11 studies were included in the review.

For the purpose of readability, we compare our results with those for the general population in order to contextualise them.

## Results

### Methods of the Studies and Risk of Bias

Eleven articles were identified (see Supplementary Table A) published between 1975 and 2016 and assessing 10 different samples of young people hospitalised in *soins-études* in psychiatry in FSEF. The types of study were heterogenous, explaining disparities in methods and results. Eight studies were academic (36, 38–44), medical theses or speciality dissertation, and three were instigated by FSEF either on its own (45), or in collaboration with INSERM (46, 47). These 11 studies generated five publications (48–52).

One study was cross-sectional, and aimed to characterise the overall population hospitalised in *soins-études* in all FSEF institutions in 1993 (47). The other 10 assessed the evolution of young people catered for in psychiatric *soins-études*: eight studies assessed the impact of care provision at discharge (36, 38–40, 43, 45, 46, 52), five analysed the evolution of the clinical condition of the young people after hospitalisation 9 months to 15 years after discharge (36, 38, 41, 44, 46).

We assessed the quality of the studies and report the risk of bias in the following parapraghs. Risk of bias for the outcome studies are detailed in the sections “Clinical assessment at discharge” and “The long-term evaluation of young people after hospitalisation in *soins-études*” (34).

The designs of the different studies were varied. Three studies had methodological support from research teams outside FSEF (36, 38, 46). The cross-sectional study by Gasquet was carried out by an INSERM research team (47). These four studies had a more rigouros methodology.

In the 10 studies exploring the evolution of young people during and after hospitalisation, evaluations were based on data collection from hospital medical files. For four of these studies the medical files were the only source of information (40, 43, 51, 52). In five studies, the research team re-contacted the young people after hospitalisation. In these follow-up studies, response rates ranged from 33 to 95% depending on the study (36, 38, 41, 44, 48). In the study by Pages, only the administrative archives and the information provided by the young people and the caregivers was collected, since the medical files had been destroyed in a fire in 1982 (36). Where possible, the author of this study presented the data from the administrative files for the 1,100 young people hospitalised in the centre, and information provided by respondents so as to provide elements of comparison of the two populations (see Table 1).

**Table 1:** Characteristics of the population admitted to soins-études in FSEF.

References		Péraud (46) and Péraud et al. (48) Pages (36)			**Condé-Diaz (38)**	**Halfon et al. (45, 49)**	**Gasquet and Choquet (47) and Gasquet et al. (50)**	**Levitchi (39) and Levitchi and Botbol (51)**	**Pinel (40)**	**Gibert (41)**	**Chandellier et al. (52)**	**Flais (43)**	**Pépin (44)**
Facility or facilities concerned		Dupré and Heuyer	Sarrailh		Arnaud	Neufmoutiers	The 6 FSEF clinics	Dupré	Dupré	Dupré	Dupré	Daguet	Daguet
Schooling provision in the facility or facilities		Final secondary and higher education^a^	Upper secondary		Vocational rehabilitation, BEP	Upper secondary	Upper secondary, higher education	Last 2 years secondary and higher education	Last 2 years secondary and higher education	Last 2 years secondary and higher education	Last 2 years secondary and higher education	Last 2 years secondary, general and management	Last 2 years secondary, general and management
Period of hospitalisation		1956–1966 (discharge)	1971–1985 (discharge)		1980–1984 (discharge)	1988–1990 (discharge)	1993	1999	1995–2004	2006 (discharge)	2007–2012 (admission)	2012–2014 (discharge)	2012–2014 (discharge)
Numbers		913	1,100	327^b^	140	109	447	20	111	43	65	63	42^c^
Mean age at admission (years)		22.3	*17.3*	*17.1*	22.4	16.8	21	20.2	18.8	19.5	18.3	18	18
Age range (years)		15–33	14–23	14–23	18–35	14–20	13–24	17–24	–	16–25	15–24	14–21	14–21
Male gender (%)		61.9	–	81^d^	67.8	56	47.9	50	4.5^e^	44	46.2	46	*39*
Profession of the father or both parents (%)	Executive	55.5^f^	18.3^g^	–	24.2^f^	*27.3*^g^	35.2^f^	–	56^f^	88.4^g^	–	54.1^f^	43^g^
	Intermediate profession	14^f^	16.3^g^	–	17.1^f^	*19.6*^g^	18.9^f^	–	16^f^	0^g^	–	} 31.8^f^	25^g^
	White collar	} 17.5^f^	14^g^	–	20^f^	*17.5*^g^	13.6^f^	–	8^f^	0^g^	–		13^g^
	Blue collar		32.2^g^	–	22.1^f^	*9.8*^g^	9.6^f^	–	7^f^	2.3^g^	–	–	7^g^
	Not working	13^f^	9.7^g^	–	1.4^f^	*18.6*^g^	13^f^	–	0^f^	4.7^g^	–	4.8^f^	3^g^
Parents married (%)		65.7	–	–	67.1	62	62.9	–	78	76.7	58.5	73	68.3
Parents separated/divorced (%)		11.2	–	–	15.7	*18*	25.5	–	19	18.7	41.5	19	21.9
Parent unknown/deceased (%)		20	–	–	12.8	*11*	11.6	–	3	4.6	0	4.8	9.8
Age of 1st contact with psychiatry (years)		–	–	–	17.3	13.7	16.5	–	–	–	–	12.3	–
History of hospitalisation in psychiatry (%)		62	–	53	94.9	75.2	(Mean *n* hospitalisations = 1.7)	–	99.1	76.7	–	90.5	87.8
History of suicide attempt (%)		–	-	–	33.5	45	25.8	–	24	32.5	55.4	44.4	46.3
Family psychiatric history (%)		37	–	–	36.4	*54.8*	70.2	–	–	58	–	58.7	63.4
Schooling difficulties	Dropout	“School mal-adaptation”: 67%	–	–	–	88% of people (>1 yr: 12.3%)	11.4% of the young people	–	–	–	–	Average 12.1 months	Average 11 months
	Delay					83.5% of young people	–		55% of the young people			–	76% of the young people
Duration of stay		7.7 m	11.5 m	14 m	17.1 m	11.2 m	9.7 m	8.8 m	16.1 m	11.5 m	18.7 m	10 m	11 m
(Standard deviation)					(±12.1)	(±9.6)	(Hospitalisation on-going)		(±13.6)	(±15.1)	(±11.9)		
(Minimum-maximum)^h^		(10 d−3 yr)	(<1 m−3 yr)		(1 m−4.5 yr)					(<1 m to >2 yr)	(23 d−4.2 yr)	(8 d−22.8 m)	(<1 m– 2 yr)

a *A small number of young people hospitalised in 4th to 6th year secondary were admitted in the first years following the switch of the Dupré clinic to psychiatry*.

b *Numbers of respondents (327) in the outcome study of the 1,100 young people hospitalised over the period*.

c *Number corresponding to the 42 respondents in the cohort of 63 young people in the Daguet clinic, Flais*.

d *Coeducation from 1981*.

e *Population of young people with anorexia*.

f *Profession of father*.

g *Profession of parents*.

h *d, days; m, months; yr, years*.

*- = NA, not available, data not collected*.

*The data in italics was calculated a posteriori from information available in the studies*.

Two studies concerned only young people with specific psychiatric disorders [psychotic disorders and emotionally unstable personalities (51); anorexia nervosa (40)], while others concerned all the young people catered for in FSEF. Consequently the studies obtained only limited results that can be generalised only to these populations.

The earliest studies (36, 38, 46) and the cross-sectional study (47) concerned the largest samples. Only two studies had multi-centre recruitment (46, 47). All the other studies were single-centre. Five of the 11 studies involved the Dupré clinic where the first psychiatric unit was opened (8). The resources mobilised, the size of the facilities and the length of hospitalisation, and thus the scope for recruitment partly explain the small sample sizes.

In all, 10 studies on the evolution of young people in the course of care were carried out in six different FSEF establishments. They covered all the age groups and all the schooling options in FSEF (mainly upper secondary, but also lower secondary and higher education, as well as vocational training in one centre).

## Population Managed in *Soins-études*

### Ages of the Young People

The characteristics of the young people taken into *soins-études* are grouped in Table 1. The mean age of the subjects included in the studies was between 16.8 and 22.4 years, most often under 20 years. Indeed, one of the main characteristics of these facilities is that they take in adolescents and young adults, often in upper secondary school. Two studies concerned older populations. The earliest was conducted in the 1970s, when there was no upper age limit for admission to *soins-études*, since the indication was based on enlistment in a higher education course The second study (38) recruited people who already had administrative recognition of their disabled status for professional rehabilitation courses.

### The Gender Ratio of Young People in *Soins-études*

The gender-ratio of the populations hospitalised in FSEF units evolved over time, and reflects the upper secondary school and higher education student profiles in France: women accounted for around 40% of the students in universities in the early 1960s (53) and for around 55% in universities and secondary schools in France today (54). The study by Pages (36) however noted a proportion of 81% boys, since the facility had only been co-educational for the last 4 years of the evaluation out of 15 years in all. The study by Condé-Diaz (38) also found a large proportion of men, probably linked to the curricula on offer, since the proportions of men in vocational training tends to be larger (55).

### Socio-Economic Backgrounds

The number of socio-economic categories and the way they were measured (profession of the father or of both parents) varied across FESF studies (see Table 1).

There appears to be an over-representation of the more privileged socio-economic categories among the young people hospitalised in *soins-études*. In France the socio-economic level of upper secondary school pupils and young people in higher education is generally above that of the general population (54). In the study by Péraud, 55% of the young people were from families in privileged socio-professional categories (46), while they were 47% in higher education in France in 1960 (56). The study by Pages concerned patients who were in upper secondary school, among whom there were 18% whose fathers were executive or managerial (36), a figure that is close to those reported in the French population in the same period (56, 57). In 2013, 30.4% of the French upper secondary school students in the general courses belonged to the upper socio-professional categories (54). The FSEF studies thus more often observed young people from the upper socio-economic categories than other facilities.

Thus, the data on socio-economic background varied because of the social heterogeneity of the territories in which the units are located, although the impact of this factor is not easy to evaluate because of the national rather than local recruitment of certain units [27–67% of extra-regional recruitment (36, 38, 43, 46, 52)]. The probability of responding to this type of survey is also greater among the more privileged social classes (58). Finally, the differences could also be linked to social biases in access to care (59), since the *soins-études* facilities are fairly specific and may not be well-known among healthcare professionals.

### Family Background

The studies also sought to assess the family backgrounds of the young people in the units (see Table 1). In the earliest study (46), at least one of the parents of 20% of the young people was unknown or deceased. The proportion is coherent with the data available for that period on national level (60) because their parents could have died, been deported or been a prisoner during Second World War.

The percentage of married parents was stable over time for the different FSEF surveys, at 60–70%, while alongside the divorce rate increased sharply in the general population nationally (61, 62). At the start of the 1960s the authors described only 65% united families (48) while in the general population the percentage of minors with parents who were married was 85% (61). In the lastest studies, the percentage of young people living in so-called “traditional” families (as opposed to single-parent or re-composed families) appeared to be more in line with FSEF populations (40, 41, 43) and the general population (62, 63), i.e., 65–70%. Nevertheless, the information collected by INSEE (national statistics institute) concerned all families, including those with young children, thus under-estimating the numbers of disunited families. The ESCAPAD population survey, which concerns young people of 17, for its part found 65% of the young people living in a nuclear family (64).

Overall, in recent years the population catered for by FSEF tended more often to be living in so-called “traditional” families, which was not the case in the earlier studies.

### Psychiatric History

The young people had been in care for more than 3 years before admission to *soins-études* (38, 43–45, 47) (see Table 1). Their disorders had required hospitalisation in more than half the cases [53–99% (36, 38, 40, 41, 43–47)]. Among the young people catered for by FSEF from 24 to 55.4% presented previous suicide attempts, while in the French population around 2.7% of young people aged 17 reported having attempted suicide (65). Further to this, in the six samples where the information was available 36.4% (38) to 70.2% (47) of the subjects in care at FSEF described a family history of psychiatric disorders. The wide variation in these proportions was linked to the type of psychiatric history taken into account, to the diagnoses or disorders requiring specialist care, and to the family members considered. These figures are in line with those found in the international literature among adolescents hospitalised in psychiatric departments (66, 67).

### Psychiatric Diagnoses

The diagnoses indicated are those retained by the teams (or psychiatrists) most often at discharge (see Supplementary Table B). The studies by Pages and Gibert (36, 41) used a clinical classification without reference to any international classification. The other studies used the INSERM nomenclature (46) then the Classification Française des Troubles Mentaux (38), the Diagnostic and Statistical Manual of Mental Disorders (DSM)—III-R (45, 47) then IV (40) and the International Classification of Diseases (ICD-10) (42–44, 51).

In the earliest study, published in 1974, Péraud (46) described 45% schizophrenic 26% neuroses and neurotic states, 14.5% “psychopathies and pathological characteristics,” 5% manic-depressive psychoses, 4.5% brief psychotic disorders, and 6% other diagnoses (anorexia nervosa, reactive depression, and mental disturbances symptomatic of epilepsy).

Pages (36) described 72% psychoses and borderline states, 35% neurotic states and 14.5% “adolescent crises” and other diagnoses. This last category grouped “reactional” suicide attempts, subjects with “pre-psychotic personalities” or presenting addictive behaviours or “minor delinquency.” The authors considered that this breakdown of disorders was identical between the 162 respondents for whom the diagnosis was known and the 1,100 young people for whom the information was retrieved from administrative archives (found despite the fire).

In the study by Condé-Diaz (38), dedicated to professional rehabilitation courses, the physicians found 87.7% with schizophrenia, 7.9% with borderline states, and 4.3% with schizophrenia and thymic disorders.

The study by Halfon (45) found 27.9% with affective disorders, 23% with anxiety disorders, 13.1% with “disruptive behaviour,” 9.3% with eating disorders and 7% with other diagnoses.

The cross-sectional study by Gasquet and Choquet (47) described 48% psychotic disorders, 28% anxious-depressive disorders, 12% eating disorders, and 12% behavioural disorders, disorders linked to substance abuse and other diagnoses (not specified in the study).

In the study by Levitchi and Botbol (51), only the young people with psychotic disorders and borderline personality disorders were included, with a sample comprising 70% schizophrenics and psychotic disorders and 30% emotionally unstable personalities.

The study by Gibert (41) found 35% with schizophrenia, 23.2% borderline personality disorders, 16.3% eating disorders, 14% mood disorders, 7% obsessive-compulsive disorders, and 4.5% hysterical personality disorders.

All the young people included in the study by Pinel (40) met the DSM-IV criteria for anorexia nervosa.

The study by Chandellier (42) found 27% with delusional disorders, 24.6% personality disorders, 23.1% eating disorders, 15.4% neurotic and somatoform disorders, 7.7% mood disorders, and 1.5% pervasive developmental disorders.

In the cohort studied by Flais (43) [from which the study by Pépin was derived (44)], the authors described 28.6% personality disorders, 25.4% psychotic disorder, 19% neurotic or somatoform disorder (including 25% obsessive-compulsive disorder), 14.3% eating disorders, 7.9% pervasive developmental disorders, and 4.8% mood disorders.

In all, on the basis of the use of international classifications, it appears that the very large majority of the young people hospitalised in *soins-études* had severe, chronic psychiatric disorders, starting in adolescence and continuing into adulthood.

### Educational Level

Certain studies set out to assess the impact of the disorders on the earlier schooling. They frequently observed schooling delays or dropout (40, 43, 45–47). Thus, three studies measured delays in schooling and found proportions ranging from 55 to 83.5% among the young people studied (40, 44, 45). These figures are very high compared to those for the French population, where the proportion of pupils who have repeated a school year is estimated to be 30% (65).

## Assessment at Discharge From *Soins-éTudes* Hospitalisation

### Length of Stays

Eight studies assessed the impact of management in a *soins-études* system at discharge (see Table 2). Hospitalisations lasted generally more than 6 months in all these studies (7.7–18.7 months). The figures were however distorted by the fact that certain studies excluded stays that were considered too short (from less than a week to <4.5 months) (38, 45, 46).

**Table 2:** Description of the young people and their evolution in studies that assessed evolution at discharge from soins-études.

References		Péraud (46) and Péraud et al. (48)Pages (36)		**Condé-Diaz (38)**	**Halfon et al. (45, 49)**	**Levitchi (39) and Levitchi and Botbol (51)**	**Pinel (40)**	**Chandellier et al. (52)**	**Flais (43)**
Facility/facilities concerned)		Dupré and Heuyer	Sarrailh	Arnaud	Neufmoutiers	Dupré	Dupré	Dupré	Daguet
Schooling provision possible in the facility or facilities		Final secondary and higher education	Upper secondary	Vocational rehabilitation, BEP	Upper secondary	Last 2 yrs secondary, higher education	Last 2 yrs secondary, higher education	Last 2 yrs secondary, higher education	Last 2 yrs secondary, general and management courses
Period of hospitalisation of the population		1956–1966 (discharge)	1971–1985 (discharge)	1980–1984 (discharge)	1988–1990 (discharge)	1999	1995–2004	2007–2012 (admission)	2012–2014 (discharge)
Numbers		913	327	140	109	20	111	65	63
Mean age at admission (years)		22.3	17.1^a^	22.4	16.8	20.2	18.8	18.3	18
Range (years)		15–33	14–23	18–35	14–20	17–24	–	15–24	14–21
Male gender (%)		61.9	81^b^	67.8	56	50	4.5	46.2	46
Age of 1st contact with psychiatry		–	–	17.3	13.7	–	–	–	12.3
History of hospitalisation in psychiatry (%)		62	53	94.9	75.2	–	99.1	–	90.5
History of suicide attempt (%)		–	–	33.5	45	–	24	55.4	44.4
Average duration of stay (months)		7.72	14	17.1	11.2	8.8	16.1	18.7	10
Clinical evaluationat discharge	Improvement (%)	63^c^	43.8^c^	52.1^d^	Improvement in global functioning (axis V DSM-III-R), *p* = 0.02	45^e^	Increase in BMI^f^ 4.2% on average	*56.9*^e^	60.3^e^
	Stagnation (%)	37^c^	40.7^c^	37.8^d^		20^e^		*30.8*^e^	39.3^e^
	Aggravation (%)		23^c^	9.2^d^		35^e^		*12.3*^e^	
Death from suicide in the course of care (%)		0.5	0.6	0.7	–	0	0	0	0
Obtaining a diploma (%)		11	–	30	30 (diploma or graduation to next level)	–	–	–	71.8 (passed the Baccalauréat in the final year secondary)

a *Mean age calculated from the graphs provided by Pages*.

b *Co-educational from 1981*.

c *Evaluation criterion: assessment by the psychiatrist in the facility*.

d *Evaluation criterion: assessment by multidisciplinary team in the facility*.

e *Improvement on the GAF (Global Assessment of Functioning): Levitchi: increase in GAF scored from medical files, Chandellier increases in GAF score of more than 25%, Flais: transition to next academic level and GAF scored from medical files*.

f *BMI, Body Mass Index: weight (kg)/[stature (m)]^2^*.

*- = NA, not available, data not collected*.

*The data in italics was calculated a posteriori from information in the studies*.

### Clinical Assessment at Discharge

The evaluation criteria to assess clinical status at the end of *soins-études* hospitalisation varied from one study to another (see Table 2). In the earliest studies, clinical improvement at discharge was assessed by the care team (36, 38, 46). Thereafter FSEF psychiatrists used scales assessing global functioning: axis V in DSM-III-R (45) and then the Global Assessment of Functioning (GAF) (42, 43, 51). Finally the study on young people with anorexia nervosa assessed the impact of hospitalisation from the evolution of their Body Mass Index (BMI) (40).

These eight studies overall found an improvement in clinical condition at discharge for 43.8–63% of the young people involved. Three studies reported clinical improvement for 60% at discharge (42, 43, 48). The studies by Pages (36), Condé-Diaz (38), and Levitchi and Botbol (51) however reported lower improvement rates (44, 45, and 52%), which should be put in perspective with the very large proportion in their samples of young people with psychotic disorders.

The three earliest studies reported deaths by suicide to be between 0.5 and 0.7% during the stay (36, 38, 48). Thus, for the period 1960–1980 the death rate by suicide was well above that reported for the French population of young people, which ranged from 8 to 24 per 100,000 among men and 5–8 per 100,000 among women (68).

### Academic Evaluation

Half of the studies set out to assess the impact of care in *soins-études* in psychiatry on academic achievement (38, 43, 45, 46). However, the evaluations of academic progress were heterogeneous. The FSEF authors recalled that academic criteria for improvement were numerous, and that they should include the resumption of school and academic effort, since a number of the young people have been out of school at admission. The study by Péraud (46) reported that 11% of their sample obtained their qualification. The authors questioned the validity of this criterion because numerous students left the facility in the course of the school year and were thus not counted as having obtained their diplomas at discharge (46). When the joint opinions of doctors and teachers were taken into account, academic results were linked to the diagnosis: where only 27% of the young people with a schizophrenic disorder obtained satisfactory results, 34% of those described as presenting “psychopathies and pathological characteristics,” while 54% obtained satisfactory results across the other diagnostic groups. The study by Halfon concerned young people from the third to the final year of secondary school. The authors described transition to the next class or passing of exams for 30% (45). For the others, 44% abandoned their schooling in the course of hospitalisation. About half of them began vocational training. The authors underlined that most of the young people in care could not have attended school outside the *soins-études* system (45). In the most recent study, 71.8% of those enlisted to sit the Baccalauréat passed.

## The Long-Term Evaluation of Young People After Hospitalisation in *Soins-études*

Five studies assessed the young subjects after their hospitalisation in *soins-études* (see Table 3). Only the most recent study used a standardised scale, the GAF, to assess the clinical status of the young people previously cared for in *soins-études* (44).

**Table 3:** Description des jeunes et de leur devenir dans les études évaluant le devenir à long terme après une hospitalisation soins-études.

References		Péraud (46) and Péraud et al. (48)Pages (36)		**Condé-Diaz (38)**	**Gibert (41)**	Pépin (44)		
Facility or facilities concerned		Dupré and Heuyer	Sarrailh	Arnaud	Dupré	Daguet		
Time lapse between hospitalisation and evaluation (years)		2–13	1–15	3–7	1	0.75–2.42		
Numbers (total/respondents)		743/913	327/1,100	140/147	43/49	42/63		
Response rates (%)		81.4	33.3	95.2	89.8	66.7		
At admission	Mean age (years)	22.25	17.1^a^	22.4	19.5	18		
	Male gender (%)	61	81 (co-ed from 1981)	67.8	44	39		
At re-contact	Mean age at evaluation	30.42	24.4^a^	28.8	20.5	–		
	Male gender (%)	63	81	67.8	44	39		
Average length of stay (months)		7.72	14	17.07	11.5	11		
Evaluation at discharge	Improvement (%)	63	43.8	52.1	–	Person: 63.4	Parents: 74.3	GAF^b^: 73.2
	Stagnation (%)	} 37	40.7	37.8	–	Person: 17.1	Parents: 17.1	GAF^b^: 26.8
	Aggravation (%)		23	9.2	–			
	Death from suicide in the course of care (%)	0.5	0.6	0.7	0	0		
Contact strategy		Letter to subject, if no reply, to parents, then GP^c^. If no reply cheque of registries	Letter to subject	Letter to subject, if no reply, to parents then to psychiatrist, then phone contacts	Phone calls to subject and parents. Letter to treating psychiatrist	Phone calls to subjects and parents. Letter to treating psychiatrist		
Type of evaluation		Interviews. If not possible interviews with parents or questionnaire to parents or GP	Questionnaires	Questionnaires	Semi-directive phone interviews with subject and parents. Questionnaire to psychiatrist	Semi-directive phone interviews with subject and parents. Questionnaire to psychiatrist		
Evaluation at re-contact	Number of death for which researchers were informed: *N* (crude mortality rate, %)	80 (*8.8*)	71 among which 22 in the fire in the facility (*21.7*)	12 (8.6)	1 (*2.3*)	1 (*2.4*)		
	Death from suicide: *N* (rate, %)	NA: “most” (of the deaths)	*≥24 (≥7.3)*	11 (7.9)	–	1 (*2.4*)		
	Hospitalisation(s) in psychiatry after discharge (%)	66.5	35	69.5	35	36.6		
	Psychiatric follow-up on-going (%)	52	33	82	62.8	63.4		
	Psychotropic treatment on-going (%)	–	27	78.9	65.1	51.2		
	Present professional activity or training (%)^d^	74.1	65	31.2	76.7	*58.5*		
	Not working (%)	21.2	35	44.2	23.2	*41.5*		
	Disability status or disability allowance (%)	11	14.5	57	7	14.6		
Clinical improvement (%)	Opinion of young people	–	65	–	–	53.7		
	Opinion of parents	–	–	–	} 63	65.7		
	Opinion of psychiatrist	72 (65% taking stabilisation into account	–	–		73.7		
	On the GAF^b^	–	–	–	–	53.7		

a *Mean age calculated from the graphs in the study by Pages*.

b *GAF, Global Assessment of Functioning*.

c *GP, general practitioner*.

d *Some had professional activity and training concurrently*.

*- = NA, not available, data not collected*.

*The data in italics was calculated from information in the studies*.

### Response Rates

Response rates ranged from 33 to 95% depending on the study. These differences were explained in particular by the means that the teams established to re-contact the young people (successive letters, phone calls, contacts with parents or contacts with the young person's psychiatrist).

### Death Rates

In all the studies, the death rates reported were high, both for overall mortality and for suicide. According to the data available in the studies, the crude mortality rate ranged from 1.3 to 2% per year, while INSEE statistics estimate mortality to be from 0.5 to 1.2‰ in the same age group (69). The study by Péraud based its mortality data on checks with official registries (46). In the other studies however the mode of collection of this information was highly likely to under-estimate the number of deaths, given the numbers lost to follow-up (5–67%). Despite this, the death rates for young people hospitalised in *soins-études* appear to be 10–20 times higher than in the general population. They also appear higher that the 0.2–0.6% death rates reported among adolescents following hospitalisation in a psychiatric facility (70–72).

Only three studies provide an estimate of death rates from suicide for these young people in *soins-études*. According to their data, the rate ranged from 1.3 to 1.7% per year (38, 44, 46). These figures appear higher than those reported for people hospitalised in psychiatric facilities in adolescence, estimated to be 0.16% per year, and then 0.56% among adults (73). This once again underlines the severity of the disorders among young people addressed to *soins-études*.

### Clinical Evolution

The number of re-hospitalisations in psychiatric departments was high, ranging from 35% at 1-year follow-up to 70% after 7 years (36, 38, 41, 44, 46). The study by Pages assessed subjects from 1 to 15 years after their period in *soins-études*. It was observed that the majority of these hospitalisations occurred in the 3 years following the hospitalisation in *soins-études* (36). The percentage of subjects with a psychiatric follow-up or receiving psychotropic treatments ranged from 30 to 82% across studies (36, 38, 41, 44, 46).

Among the respondents, 60–75% were in training or were in a job at the time of the survey (36, 41, 44, 46). Some authors linked professional activity to the types of disorder presented by the young people, observing that those with the most severe pathologies were less frequently in employment (36, 38, 46). The authors of the earliest study also found a link between the educational level or the diploma obtained and the likelihood of having a job (46). The percentage of subjects receiving specific allowances for adult disabled people ranged from 7 to 15% (36, 41, 44, 46). The percentage was reaching 57% in the study by Condé-Diaz (38) in which the population mainly comprised young adults with schizophrenic disorders, some of whom had already been oriented for professional reassignment by the French body in charge of disabled workers. In the most recent study, 41.5% had no professional activity and no training (44). In comparison, in the general population, the percentages of vocational reassignment and failure to re-enlist in university by students 1 year after the Baccalauréat are 11.1 and 25.5%, respectively (74).

In studies on outcomes, the percentage of young people considered to have improved following their stay in *soins-études* ranged from 54 to 74% (36, 41, 44, 46) (see paragraph “Clinical evaluation at discharge” for the criteria used to assess improvement).

The three studies that evaluated improvement during hospitalisation and after discharge using the same criteria (36, 44, 46) showed that the respondents maintained or pursued their recovery after discharge. Thus, in the most recent study (44), only 9.8% of the young people described an aggravation after discharge (while one subject corresponding to 2.4% of the sample did not answer the item).

## Discussion

Since the start of *soins-études* in psychiatry, FSEF teams have taken care to assess the outcomes of the young people they have managed so as to determine the impact of this approach to care. The main objective of our review of the literature was to collate all the studies conducted in order to assess the *soins-études* facilities in psychiatry since the opening of the first of them in 1956. Eleven quantitative studies on these facilities in France were found. Although they are heterogeneous, these evaluations do seem to indicate that these multidimensional interventions have a favourable impact on recovery and socio-professional rehabilitation of the young people having attended.

The young people receiving treatment in *soins-études* were mostly aged 16–20, which is the expected age group given the courses available in most of these facilities. Indeed, most enable schooling in general education courses in upper secondary school, while a few offer lower secondary school curricula. The evolution of the gender ratio observed in favour of girls is in line with access to these upper secondary courses in the general French population.

The proportion of young people from more privileged socio-economic backgrounds was more marked in the FSEF facilities than in the general courses in upper secondary school in France. However, several factors temper this result. First of all the probability of responding to surveys of this sort is greater in the more privileged social classes (58). Next, although the financial situation of families does not restrict access to *soins-études*, since hospitalisation is covered by the Sécurité Sociale, there may be bias in access to care (59). In addition, as the *soins-études* facilities are few and rather specific, they may not be well-known by certain professionals or families.

These young people receiving treatment in *soins-études* presented various severe and chronic psychiatric disorders, mainly schizophrenic disorders, affective disorders, personality disorders, autism spectrum disorders and eating disorders. The diagnostic classifications used in these studies evolved over the years, along with the international classifications. The young people in the FSEF facilities generally had chronic disorders, and their previous care trajectories were often lengthy and characterised by one or several self-harm episodes. The suicide rate, higher than in the general population, occurring during or after their stay in *soins-études*, reflects the severity of their disorders. Their clinical condition did however need to be compatible with a form of schooling to warrant admission and ensure continuity.

The length of stay in these facilities averaged more than 6 months, with a maximum of 2–4 years. As in many countries, these lengths of stay in therapeutic residential care raise concerns about difficulties in leaving the facility at discharge (17, 20, 29), and the iatrogenic effect of such long periods of care (5, 8, 20, 28, 75). Certain strategies were progressively set up to enable better continuity with the outside during and at the end of their stay. Thus, each young person was encouraged to maintain links with a reference psychiatrist outside the facility and independent from any issues relating to schooling (17, 19–21, 76). Likewise, psychotherapeutic work with the families was given prominence (18, 19).

Depending on the study, 44–63% of the young people were considered to have improved at discharge. The majority had a schooling activity during their period of treatment and were pursuing their studies. The percentages varied according to the type of disorder (the difficulties were greater for subjects with schizophrenic disorders). For the young people who did not return to school, their period in *soins-études* provided scope for the elaboration of an alternative plan. The contribution of *soins-études* appeared particularly valuable for these young people. They did indeed present severe disorders, but the evolution was favourable for 54–74% of them 1–15 years after their hospitalisation, with school attendance and plans for further training.

In acute psychiatric hospitalisation, the length of stay is significantly longer for people with more previous admissions or with severe mental illness such as schizophrenia or mood disorders (77). Among adolescents with chronic disorders, lasting acute hospital care can be damaging for social reintegration. The *soins-études* system enables social and school reintegration while at the same time providing healthcare. This is why *soins-études* is considered as third-level care and recommended in French guidelines for adolescents and young adults with psychiatric disorders (31–33).

Therapeutic residential care is now being called into question for reasons of funding (4). This raises the question as to whether this type of facility has a future in France (4). They are thus beginning to be evaluated at international level, but the studies explored programmes that were extremely heterogeneous. Indeed, the terms “residential care” cover various types of care involving health, but also child welfare with socio-educational or judicial establishments (4). In contrast, the FSEF gathers facilities where the care is prolonged and multidimensional for young people requiring mental healthcare. They therefore offer a homogenous set of residential care facilities, which to our knowledge is unique in France on this scale. Therapeutic residential care assessed in other countries is either public or private, and the establishments present differences from one to another, and with the FSEF *soins-études* system.

In international public residential care systems, the patients are generally younger (children or young teens) and more frequently male than in the populations cared for in FSEF (78–80). Behavioural disorders are the first motive for admission (78, 79, 81). The children have previously frequently been placed in other structures or in foster families, and have frequently had dealings with childhood protection units for a history of neglect, ill treatment or sexual abuse (78–81). The mean IQ of these young people is generally below average (78–81) and they have frequently had dealings with the legal system (78, 81). They often come from underprivileged backgrounds and only a minority are living with both biological parents (78, 80). Finally, these young people have often been hospitalised in the past (81) and admission to residential care is indicated as an alternative to prolonged hospitalisation in psychiatric ward. However, the admission to residential care is often decided by childhood protection bodies (78, 80) while in FSEF *soins-études* the young people and their parents apply for admission.

The meta-analyses on the efficacy of public residential care include studies that were mostly conducted in the USA and Europe (and did not include France). They noted a decrease in symptoms among the young people in care, although the improvement appeared to wane after discharge (82–84). Favourable results in the long term seemed in particular linked to the stability of accompaniment in the ambulatory facilities after discharge, and to whether or not the families were implicated in the care (82, 83). However, as this treatment is perceived as a “last resort” solution, the studies included in these reviews were only pre-experimental (two measurements over time performed within a sample, before and after an intervention) or quasi-experimental studies (two groups are offered a different intervention and studied at two time points) (84). In addition, these results provide information on the value of long-term care, but they are mainly focused on young people with behavioural problems (84), so that they do not concern systems like the one explored here.

In the USA there are also private residential care facilities, where the population intake and the functioning differ markedly from public residential care. The young people are admitted at their own request and that of their families (85, 86). They are most often oriented by an educational consultant (87, 88) for serious mental disorders, behavioural disorders (aggressive behaviours towards others), or relational difficulties with their families and/or at school (87). These facilities are closer to the FSEF facilities, since they offer environment-based therapy with individual psychiatric care and in groups, in particular *via* therapeutic activities, and family therapies (87), occurring within full-time care lasting 10–12 months on average (85, 87). The young people catered for are more often adolescents and young adults (16 years on average for adolescent facilities, 21 for the young adult facilities) (85–88), having already been in psychiatric care (85, 88), and from privileged backgrounds (given the cost for the families or their private insurance policies in the USA) (85, 87, 88). Their educational level is described as acceptable or good (88, 89), but on average they have a delay of one semester in their schooling (89). However, the profiles of the young people differ from those in FSEF facilities. They are more often boys (55–68%) (85), they more frequently present addictive pathologies or comorbidities and rarely autistic or psychotic disorders (86, 87). They also have frequently had dealings with the legal system (85, 88).

The private residential care facilities also have similarities in functioning with French *soins-études*. Indeed the programmes concerning young adults (over 18) are oriented towards fostering the autonomy of the young people, allowing them to go out freely during the day to attend training outside the facility (86). The authors that assessed these private facilities noted a marked improvement of the psychopathology, as assessed by the subjects and their families, between the start and the end of care (85). One year after discharge, symptoms had increased, but remained clearly lower than at admission, and scores were within the normal range (85). The relationships of the young people with their families and their functioning in school followed the same trend of improvement (89). Among the young adults, psychosocial and family functioning also appeared to improve in the course of treatment, an improvement that appeared to persist at 6 months (86). However, the response rate in these studies was under 30% at 1 year (85, 89).

Besides these residential care facilities, a quasi-experimental study in the USA showed the efficacy of supported education in the inpatient treatment of young adults (90). Thus, when medium- to long-term inpatient care is required, these young adults benefited from academic involvement and were significantly more likely to return to college and progress to full-time status after discharge (90). The conception behind this program was close to the conception underpinning *soins-études*, since it focused on integrating the existence of a mental illness and the need for continuing treatment in the constellation of activities and developmental processes. The authors assumed that combining effective psychotherapeutic modalities with systematic attention to strengthening functional and social skills offers more potential than the more traditional focus on symptom amelioration (6, 90).

### Limitations of the Study and Perspectives

The conclusions of our review are limited by the poor quality of the existing studies. This type of study is certainly less productive than a randomised trial, but in the words of Craig at al., experimental designs are preferred to observational designs in most circumstances, but are not always practicable (91). The work on FSEF *soins-études* facilities was often restricted to a single facility. Only one study included all FESF facilities. In addition, certain studies had selection biases, in particular because of their sampling methods. For instance only young people with specific pathologies were included (40, 51), or young people whose stay was considered too short were excluded (38, 45, 46). The studies reported on data gathered between 1956 and 2014, amounting to a large time span in which diagnostic classification systems have altered and much may have changed in therapeutic interventions as in all psychiatric institutions in the same period. Nevertheless, the *soins-études* units are still meeting a need and their number increased from 1 to 11 establishments between 1956 and 2019.

As there was only open studies evaluating *soins-études*, one could argue that the improvement observed might be the results of ageing. Future studies will need to include a control group in order to overcome this limitation. In addition, response rates in these studies were sometimes low. Only 5 of the 11 studies were published in peer-reviewed journals. This is only the first step in a *soins-études* evaluation and we need to develop other evaluations. These studies lacked standardised evaluations to establish diagnoses, their impact and the clinical evolution. Past research has not enabled the diagnosis of the disorders in subgroups, and therefore do not relate their findings to the disorders present. This makes it difficult to differentiate whether adolescents across all the different diagnostic groups are able to benefit from *soins-études*, or only a specific subgroup. Finally, only the earliest study (48) established a reliable mortality rate (checking vital status using national registries).

Given these limitations, future research needs to be prospective and to include the complete population intake. It also need to adopt mixed methods assessing not only diagnoses, impact and clinical evolution (admission, discharge and follow-up) (85, 92), but also overall outcomes (social and relational, academic or professional). In addition, these elements need to be completed by qualitative studies reporting on the opinions of young people, their parents and professionals upstream and downstream, on the contributions of this type of care. Even if an experimental design would be preferable to an observational design, it would not be practicable because of the difficulty in forming a control group (91). Therefore, future studies should opt first for a pre-post methodology (pre- or quasi-experimental design) (85, 92, 93), and they should establish diagnoses in standardised manner and assess the impact and clinical evolution using standardised multidimensional criteria, taking into account both the clinical state with the reduction in symptoms, and global, social and relational, academic and professional functioning among the young people (92, 93). Long-term mortality should be calculated using national INSEE data on deaths. Furthermore, cost effectiveness studies should be carried out because of the high cost of this type of care.

## Conclusions

The data we have appears to support the usefulness of *soins-études* in psychiatry. Nevertheless, these first evaluations are only partial. Studying systems of this sort amounts to assessing complex systems (91). Future research needs to be developed in order to improve knowledge about the efficacy of this type of care. In order to overcome the limitations of the existing studies we plan to develop a large study with a comparison group (people who have been addressed to *soins-etudes* but were not admitted).

## Data Availability Statement

The original contributions presented in the study are included in the article/Supplementary Material, further inquiries can be directed to the corresponding author.

## Author Contributions

FH and NG performed the selection process for the systematic review. FH wrote the paper. CH, DD, and NG contributed to the critical revision of the manuscript. All authors contributed to the article and approved the submitted version.

## Conflict of Interest

The authors declare that the research was conducted in the absence of any commercial or financial relationships that could be construed as a potential conflict of interest.
