# Supplementary material for: The Evaluation of Therapeutic Residential Care for Adolescents and Young Adults in France: A Systematic Review
Source: Front Psychiatry. 2021 May 20;12:609365. doi: 10.3389/fpsyt.2021.609365 (PMC8173203; doi:10.3389/fpsyt.2021.609365)
Supplement: Supplementary file 1 [file Table_1.docx]

Table A: Studies evaluating FSEF soins-études facilities according to year of recruitment of their populations

| Authors (references)) | | Péraud  (46,48) | Pages  (36) | Condé-Diaz  (38) | Halfon  (45,49) | Gasquet (47,50) | Levitchi  (39,51) | Pinel (40) | Gibert (41) | Chandellier  (42,52) | Flais  (43) | Pépin (44) |
| --- | --- | --- | --- | --- | --- | --- | --- | --- | --- | --- | --- | --- |
| Facility or facilities concerned | | Dupré and Heuyer  (Hauts-de-Seine and Paris) | Sarrailh  (Landes) | Arnaud  (Val d'Oise) | Neufmoutiers  (Seine et-Marne) | The 10 FSEF facilities including 6 involved in psychiatry | Dupré  (Hauts-de-Seine) | Dupré  (Hauts-de-Seine) | Dupré  (Hauts-de-Seine) | Dupré  (Hauts-de-Seine) | Daguet  (Sarthe) | Daguet  (Sarthe) |
| Study objectives | Evolution at discharge | X | X | X | X | Cross-sectional study in the course of care | X | X |  | X | X |  |
|  | Evolution after hospitalisation in *soins-études* | X | X | X |  |  |  |  | X |  |  | X |
| Period of hospitalisation of the population | | 1956-1966 ^[[1]](#footnote-1)^ | 1971-1985 ^1^ | 1980-1984 | 1988-1990 | 1993 | 1999 | 1995-2004 | 2006 | 2007-2012 | 2012-2014 ^1^ | 2012-2014 ^1^ |
| Year of post-hospitalisation evaluation | | 1968-1973 | 1986 | 1987 | ̶ | ̶ | ̶ | - | 2007 | ̶ | ̶ | 2015 |
| Time lapse between hospitalisation and evaluation (years) | | 2-13 | 1-15 | 3-7 | ̶ | ̶ | ̶ | - | 1 | ̶ | ̶ | 0.75-2.42 |
| Numbers (respondents/total) | | 743/913 | 327/1100 | 140/147 | 109/109 ^[[2]](#footnote-2)^ | 447/447 ^[[3]](#footnote-3)^ | 20/20 ^2^ | 111/111 ^2^ | 43/49 | 65/65 ^2^ | 63/63 ^2^ | 42/63 ^[[4]](#footnote-4)^ |
| Response rate in outcome studies (%) | | 81.4 | 33.3 | 95.2 | - | - | - | - | 89.8 | - | - | 66.7 |

Table B: Psychiatric diagnoses described among the young people hospitalised in FESF soins-études

| Authors (references) | Péraud  (46,48) | Pages  (36) | Condé-Diaz  (38) | Halfon  (45,49) | Gasquet (47,50) | Levitchi  (39,51) | Pinel (40) | Gibert (41) | Chandellier  (42,52) | Flais  (43) | Pépin (44) |
| --- | --- | --- | --- | --- | --- | --- | --- | --- | --- | --- | --- |
| Facilities | Dupré and Heuyer | Sarrailh | Arnaud | Neufmoutiers | The 6 FSEF clinics | Dupré | Dupré | Dupré | Dupré | Daguet | Daguet |
| Period of hospitalisation | 1956-1966 ^[[5]](#footnote-5)^ | 1971-1985 ^1^ | 1980-1984 | 1988-1990 | 1993 | 1999 | 1995-2004 | 2006 | 2007-2012 | 2012-2014 ^1^ | 2012-2014 ^1^ |
| Numbers | 743/913 | 327/1100 | 140/147 | 109/109 ^[[6]](#footnote-6)^ | 447/447 | 20/20 ^2^ | 111/111 ^2^ | 43/49 | 65/65 ^2^ | 63/63 ^2^ | 42/63 ^[[7]](#footnote-7)^ |
| Diagnostic classification used ^[[8]](#footnote-8)^ | INSERM diagnostic nomenclature | Not defined | CFTM | DSM-III-R | DSM-III-R | ICD 10 | DSM IV | Not defined | ICD 10 | ICD 10 | ICD 10 |
| Diagnoses described | 45% schizophrenia  4.5% acute psychoses  5% manic depressive psychoses  26% neuroses  14.5% psychopathies and pathological features  6% other diagnoses (anorexia nervosa, reactive depression and mental disorders linked to epilepsy) | 72% psychoses and borderline states (71% of the total population) ^[[9]](#footnote-9)^  13.5% neurotic structure (13%)  14.5% adolescent crises and other diagnoses (pre-psychotic personality, addictive behaviours, "minor delinquency") (16%) | 87.7% schizophrenia  7,9% borderline states  4.3% schizophrenia with thymic disorders | *19.7*% psychotic disorders  *27,9*% affective disorders  *23*% anxiety disorders  *13.1*% disruptive behaviours  *9.3*% eating disorders  *7*% other diagnoses | 48% psychotic disorders  28% anxious-depressive disorders  12% behavioural disorders linked to substance use and other diagnoses  12% eating disorders | 70% schizophrenia and psychotic disorders ^[[10]](#footnote-10)^  30% emotionally labile personalities ^6^ | 100% anorexia nervosa ^6^ | 35% schizophrenia  23.2% borderline personality disorder  14% mood disorders  7% obsessive-compulsive disorders  4.5% hysterical personality disorder  16.3% eating disorders | 27.7% delusional disorders  7.7% mood disorders  15.4% neurotic and somatoform disorders  24.6% personality disorders  1.5% pervasive developmental disorder  23.1% eating disorders | 25.4% psychotic disorders  4,8% mood disorders  19% neurotic or somatoform disorders (incl. 25% of obsessive compulsive disorders)  28.6% personality disorders  7.9% pervasive developmental disorders  14.3% eating disorders | 26,9% psychotic disorders  7,3% mood disorders  17,1% neurotic or somatoform disorders  21,9% personality disorders  4,9% pervasive developmental disorders  21,9% eating disorders |

1. First years of functioning of the facility concerned or first years following the opening of the first psychiatry beds [↑](#footnote-ref-1)
2. Study collecting data from medical files and including all young people meeting the inclusion criteria [↑](#footnote-ref-2)
3. 447 young people hospitalised in psychiatric department of the 1069 included in all the FSEF facilities (622 hospitalised in somatic medicine) [↑](#footnote-ref-3)
4. Number corresponding to 42 respondents in the cohort of 63 young people in the Daguet clinic cohort, Flais [↑](#footnote-ref-4)
5. First years of functioning of the facility concerned or first years following the opening of the first psychiatry beds [↑](#footnote-ref-5)
6. Study collecting data from medical files and including all young people meeting the inclusion criteria [↑](#footnote-ref-6)
7. Number corresponding to 42 respondents in the cohort of 63 young people in the Daguet clinic cohort, Flais [↑](#footnote-ref-7)
8. INSERM : Institut National de la Santé Et de la Recherche Médicale ; CMTA : Classification Française des Troubles Mentaux ; ICD : International Classification of Diseases; DSM : Diagnostic and Statistical Manual of Mental Disorders [↑](#footnote-ref-8)
9. Percentage in the population of respondents (percentage in the total population of 1100 young people included in the study over the period) [↑](#footnote-ref-9)
10. Diagnoses included in the study inclusion criteria

    *The data in italics was calculated a posteriori from information available in the studies* [↑](#footnote-ref-10)
